# Taking the brakes off telomerase

**DOI:** 10.7554/eLife.09519

**Published:** 2015-07-21

**Authors:** Kamila Naxerova, Stephen J Elledge

**Affiliations:** Department of Genetics, Harvard Medical School; and the Division of Genetics, Brigham and Women's Hospital, Boston, United States; Department of Genetics, Harvard Medical School; the Howard Hughes Medical Institute; and the Division of Genetics, Brigham and Women's Hospital, Boston, United Statesselledge@genetics.med.harvard.edu

**Keywords:** telomerase TERT, immortalization, cancer mechanism, genome editing, tumor spectrum, ssociated systems 9 CAS9, human

## Abstract

Studies using human embryonic stem cells have revealed how common cancer-associated mutations exert their effect on telomerase after cells differentiate into more specialized cell types.

**Related research article** Chiba K, Johnson JZ, Vogan JM, Wagner T, Boyle JM, Hockemeyer D. 2015. Cancer-associated TERT promoter mutations abrogate telomerase silencing. *eLife* **4**:e07918. doi: 10.7554/eLife.07918

**Image** Stem cells with TERT promoter mutations can still differentiate into more specialized cells such as nerve cells

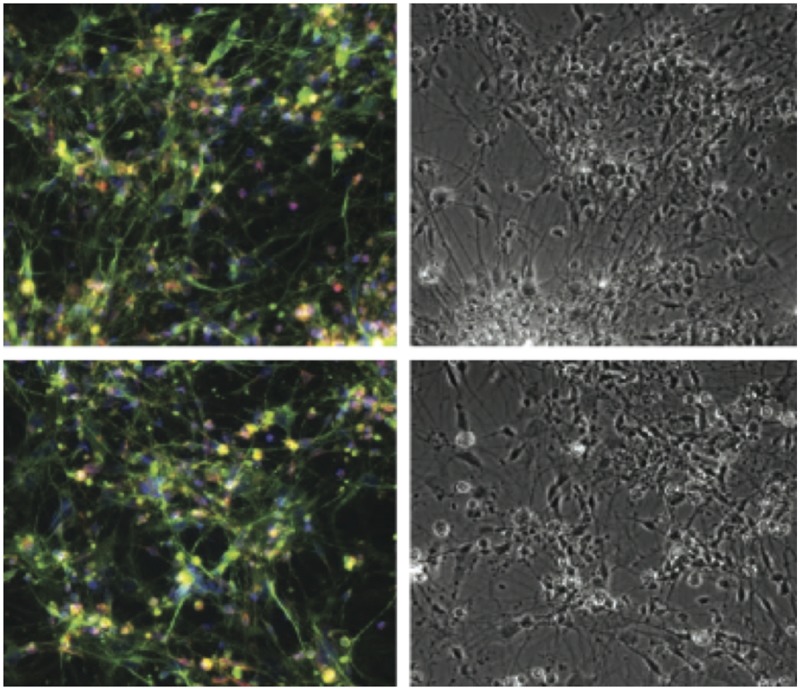

Most cells in the human body have a limited lifespan. This is because a cell loses a small portion of each of its telomeres—the DNA sequences that protect the ends of chromosomes—every time it divides. Once the telomeres fall below a critical length, which usually happens after approximately 40–60 cell divisions, the cell dies or enters a permanent state called senescence and no further divisions take place.

To avoid this fate, stem cells express an enzyme called telomerase, which can elongate and maintain telomeres. The enzyme contains a molecule of RNA and an active protein component called TERT. Telomerase activity essentially makes stem cells immortal, which allows them to replenish the tissues in an organism over its lifetime. However, this ability is lost when stem cells undergo a process called differentiation and change into more specialized cell types.

Like stem cells, cancer cells must maintain their telomeres to grow continually. Recently, researchers found an important clue as to how cancers may achieve this. Two independent groups identified recurring mutations in the promoter region of the gene that encodes TERT (Horn et al., 2013; Huang et al., 2013). These mutations occur with very high frequency in some tumor types. For example, approximately 80% of glioblastomas (the most common and aggressive type of brain tumor in humans) contain TERT promoter mutations, as do 80% of liposarcomas and 60% of bladder cancers (Killela et al., 2013; Vinagre et al., 2013). In fact, researchers now believe that TERT promoter mutations may be the most prevalent mutations of all in some tumor types. Unexpectedly, some common cancers, like breast and colon carcinoma, do not harbor TERT promoter mutations. This finding indicates that the selective advantage of these mutations varies from tissue to tissue.

The next crucial step was to elucidate how TERT promoter mutations might contribute to cancer. Now, in *eLife*, Dirk Hockemeyer and colleagues at the University of California, Berkeley—including Kunitoshi Chiba as first author—report progress in this direction (Chiba et al., 2015). Previous studies that evaluated the effects of TERT promoter mutations on TERT expression levels and telomerase activity in cancer cell lines had found relatively modest differences between mutant and wild type cells (Horn et al., 2013; Huang et al., 2013; Borah et al., 2015). However, by definition, all cancers must have already found a way to maintain their telomeres. Chiba et al. therefore decided to study TERT promoter mutations in normal human embryonic stem cells, both before and after they differentiated into more specialized cells. The Berkeley team used genome editing to create four stem cell lines that were genetically identical except for small differences in the TERT promoter region; one cell line contained the wild type sequence and the others carried one of the three mutations commonly observed in cancer. In line with previous reports, Chiba et al. saw only minor differences in the level of TERT expression between the wild type and mutant cells. However, closer scrutiny revealed that, in contrast to more mature cell types, the expression level of the TERT gene cannot be used to predict telomerase activity in stem cells. This is because, in these cells, telomerase is limited by a shortage of the RNA component, and not by a shortage of the TERT component.

Instead, the crucial insight emerged when Chiba et al. differentiated the stem cells into nerve cells and fibroblasts (cells commonly found in connective tissue). While the expression of TERT was down-regulated in wild type cells, it was not down-regulated in the cells with mutations. This led to strong telomerase activity in the cells with mutations, which allowed them to maintain longer telomeres (Figure 1). Furthermore, telomerase activity in the differentiated cells with TERT promoter mutations was very similar to the level of activity in cancer cell lines, which further illustrates the powerful effects of these promoter mutations. Finally, the Berkeley team showed that human cells with TERT promoter mutations maintain longer telomeres when they are transplanted in mice.

**Figure 1.**
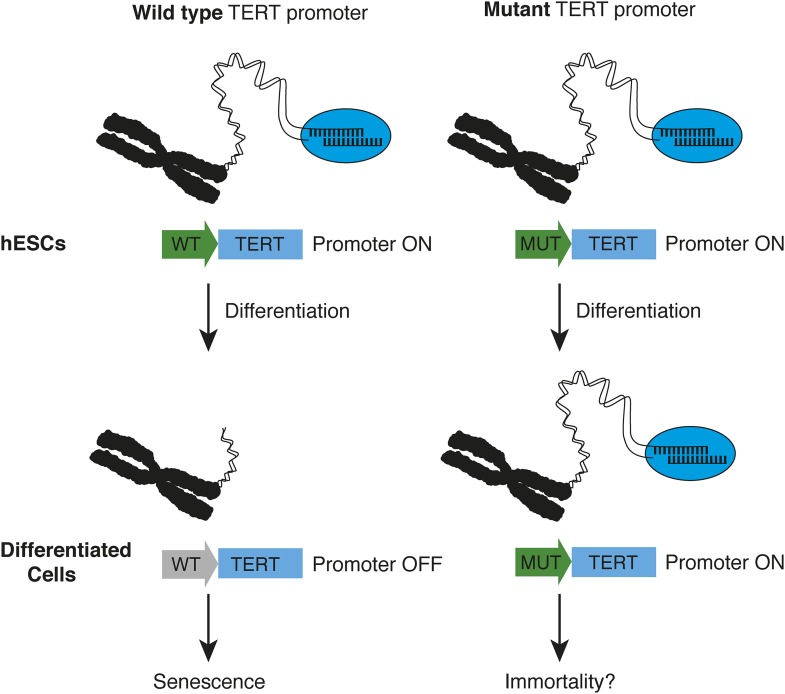
How TERT promoter mutations may contribute to cancer. Chiba et al. report that in human embryonic stem cells (hESCs, top), the promoter (green arrow) of the telomerase gene (TERT) is active, regardless of whether it is wild type (WT, left) or mutated (MUT, right). The telomerase enzyme (blue ellipse) maintains long telomeres at the chromosome ends. When the stem cells differentiate into fibroblasts or nerve cells (bottom), telomerase expression is appropriately down-regulated in cells with a wild type TERT promoter (grey arrow), and telomeres begin to shorten—which leads to senescence. However, this does not occur when cells with telomerase promoter mutations differentiate—which may allow the cells to become immortal.

The prevalence of TERT promoter mutations suggests that they confer a strong selective advantage, but this seemed somewhat at odds with the relatively small change in TERT expression that is caused by these mutations. The results of Hockemeyer, Chiba and colleagues—which show that differentiated cells with TERT promoter mutations can circumvent the down-regulation of telomerase—now help to explain the prevalence of these mutations.

Nonetheless, much remains to be learned about the role of TERT promoter mutations in the formation of cancer. For example, is this level of telomerase expression sufficient for immortality? In addition, it is still poorly understood why these mutations occur in such a tissue-specific manner. Chiba et al., as well as others (Killela et al., 2013), rightly suggest that these mutations may be more important in tissues that do not self-renew at a high rate and have few telomerase-expressing cells. However, prominent exceptions to this paradigm exist. For example, the lining of the inside of the mouth is continuously self-renewing, but carcinomas of the oral cavity frequently harbor TERT promoter mutations (Killela et al., 2013). A careful analysis of the transcription factors that can bind the mutant TERT promoter (like GABP for example (Bell et al., 2015)) and the tissue-specific expression patterns of these transcription factors may add a missing piece to this puzzle. The prevalence of TERT promoter mutations, their intuitively obvious importance for cancer development and progression, and the resulting therapeutic potential, will undoubtedly unleash significant efforts to answer these questions in the near future.
